# Evaluating the Y chromosomal STR dating in deep-rooting pedigrees

**DOI:** 10.1186/s13323-015-0025-z

**Published:** 2015-05-28

**Authors:** Chuan-Chao Wang, Hui Li

**Affiliations:** State Key Laboratory of Genetic Engineering and Ministry of Education Key Laboratory of Contemporary Anthropology, Collaborative Innovation Center for Genetics and Development, School of Life Sciences, Fudan University, Shanghai, 200433 China

**Keywords:** Y chromosome, STR, Mutation rate, Time estimation

## Abstract

**Background:**

Y chromosomal short tandem repeat (STR) has been used in time estimations for single nucleotide polymorphism (SNP) lineages or eminent persons. But to choose which mutation rate and estimation method in the Y chromosome dating is controversial, since different rates and methods can result in several-fold deviation.

**Findings:**

We used two deep-rooting pedigrees with full records and reliable dates to directly evaluate the Y chromosomal STR mutation rates and dating methods. We found that the Y chromosomal genealogical mutation rates (OMRB and lmMR) in BATWING method can give the best-fit estimation for historical lineage dating.

**Conclusions:**

This study validated a very efficient and reliable way for genealogy and historical anthropology researches.

**Electronic supplementary material:**

The online version of this article (doi:10.1186/s13323-015-0025-z) contains supplementary material, which is available to authorized users.

## Findings

The paternally inherited Y chromosome has been proved to be a superb tool in inferring human population demographic history, forensic identification, and genetic genealogy [1]. There are two kinds of extremely useful markers in Y chromosome, single nucleotide polymorphism (SNP) and short tandem repeat (STR). With a very low mutation rate on the order of 3.0 × 10^−8^ mutations/nucleotide/generation [2], SNP markers have been used in constructing a robust phylogeny tree linking all the Y chromosome lineages from world populations [3, 4]. However, the mutation rates of STRs are about 4 to 5 orders of magnitude higher than SNPs [5]. The high mutation rates of STRs make them extremely useful in forensic identification and population diversity estimation. The most important link between genetic diversity and human history is time, for instance, the time when a lineage originated or expanded or when a population split from another and migrated. Y-STR has also been used in time estimations for SNP lineages or eminent persons [1]. The well-known example was the determination of Genghis Khan’s lineage [6]. Although this approach is widely used, there are still many ongoing debates about the best way to use STRs in lineage dating. In particular, there are two popularly used Y chromosomal STR mutation rates, that is, the genealogical rate and the evolutionary rate. The genealogical rates are directly observed rates in father-son pairs [7]. The evolutionary rates are those calibrated against historical events, such as the divergence of the Maoris and Cook Islanders in the Pacific [8]. There are also two widely used methods in Y chromosomal STR dating, average squared distance (ASD) [9–15] and Bayesian analysis of trees with internal node generation (BATWING) method [16]. ASD method is based on the assumption that median or modal STR haplotype in a lineage is the founder haplotype [11–15]. BATWING uses a Markov chain Monte Carlo (MCMC) method based on coalescent theory to generate approximate random samples from the posterior distributions of parameters [16]. To choose which mutation rate and estimation method in the Y chromosome dating is controversial, since different rates and methods can result in several-fold deviation.

Recording the genealogy has been a tradition of Han Chinese, and some genealogical trees even link the contemporary individuals to their ancestors over 2000~3000 years ago, which has provided the best approach to evaluate the Y chromosomal STR dating. Here, we collected two pedigrees with full records claimed to be descendants of Duke Zhao Shi (A.D. 1057–1129) [17] and Miaorong Cào (A.D. 1341–1411) [18]. We collected blood samples from 4 male individuals of Shi clan and 14 male individuals of Cào clan. In the two pedigrees studied here, 28 meiotic events happened between Duke Zhao Shi and his latest descendant and 21 meiotic events happened between Miaorong Cào and his latest descendant. The study was under the approval of the Ethics Committee of Biological Researches at Fudan University, and all the samples were collected with the Informed Consents. For all these samples, we extracted DNA, typed phylogenetic relevant Y chromosomal SNPs as listed in the latest Y-chromosomal tree as we did in previous studies [19, 20], and amplified 17 Y-STRs (DYS19, DYS389I/II, DYS390,DYS391, DYS392, DYS393, DYS437, DYS438, DYS439, DYS448, DYS456, DYS458, DYS635, Y-GATA H4, and DYS385a/b) using Y-Filer kit (Life Technologies, Carlsbad, CA, USA). Shi clan has been identified as haplogroup O1a1-P203 and Cào clan has been assigned as haplogroup O3a2c*-P164+, M134- (Additional file 1). Time estimation for each Y chromosomal lineage was made using both ASD and BATWING method based on 15 STRs (excluding DYS385a/b). The ages in ASD were calculated within our contemporary samples by comparing to the modal and median haplotype. It is worthy to mention that the median and modal haplotypes are the same in the two pedigrees at the used 15 loci. Four sets of Y-STR mutation rates [7] were applied in the estimations (Additional file 2). These are a widely used evolutionary mutation rate (EMR) [8], two observed genealogical mutation rates (OMRB and OMRS) [21, 22], and a genealogical mutation rate adjusted for population variation using logistic model (lmMR) [21]. A generally accepted generation time of 25 years was used to produce a time estimate in years. Through cross-cultural estimation, Fenner proposed a male generation length of 31–32 years [23], which were also used for comparison. For BATWING method, we used a model of exponential growth from an initially constant-sized population. We applied weakly informative prior distributions parameters in BATWING estimations to avoid possible biases caused by parameter changing. For the initial effective population size (*N*), we used a broad prior gamma (1, 0.0001) (mean = 10,000, SD = 10,000). For population growth rate per generation (*α*), we also used the broad prior distributions gamma (2, 400) (mean = 0.005, SD = 0.0035). The time in coalescent units when exponential growth (*β*) began was used gamma (2, 1) (mean = 2, SD = 1.41) [24]. A total of one million MCMC samples were collected per run in BATWING, and the first 3000 were discarded as burn-ins. The time to the most recent common ancestor (TMRCA) is calculated using the product of the estimated population size *N* and the height of the tree *T* (in coalescent units).

The results were given in Table 1. Shi clan in this study can trace their common ancestor to Duke Zhao Shi 885–957 years ago (ya) and Cào clan can trace their ancestor to Miaorong Cào 603–673 ya. The BATWING method applying genealogical mutation rates, OMRB and lmMR, gave the best-fit estimations, 859.7 and 887.0 ya for Duke Zhao Shi and 671.1 and 704.5 ya for Miaorong Cào. The OMRS rate in BATWING has underestimated the time for Shi and Cào at about 200~270 and 70~140 years shorter, respectively. However, the estimates using evolutionary mutation rate are 3~4 times larger than the real time. On the contrary, the ASD method using genealogical mutation rates has given the results 3~4 times smaller than the real time. While applying evolutionary rate, ASD gives 1207.7 and 1035.2 ya for Shi and Cào, respectively, although quite near their real living time but still has 200~300-year deviation. We changed the generation time to 31~32 years as Fenner proposed [19] and also got very similar results.

**Table 1:** Time estimation in two deep-rooting pedigrees using both BATWING and ASD method (time in years)

	Clan	OMRB		OMRS		lmMR		EMR	
		TMRCA	95 % CI	TMRCA	95 % CI	TMRCA	95 % CI	TMRCA	95 % CI
BATWING	Shi (885~957 ya)	859.7	199.5–3543.6	686.2	154.7–2895.3	887.0	241.9–2577.7	3424.5	833.1–14,101.0
	Cào (603~673 ya)	671.1	211.5–1833.5	534.0	165.1–1480.0	704.5	222.2–1925.2	2658.4	840.6–7957.9
		TMRCA	SE	TMRCA	SE	TMRCA	SE	TMRCA	SE
ASD	Shi (885~957 ya)	315.9	417.3	242.7	342.2	379.7	501.0	1207.7	1593.595
	Cào (885~957 ya)	237.1	121.9	221.9	115.1	206.2	101.1	1035.2	509.61

In this study, we used two deep-rooting pedigrees with full records and reliable dates to directly evaluate the Y chromosomal STR mutation rates and dating methods. We found that the Y chromosomal genealogical mutation rates (OMRB and lmMR) in BATWING method can give the best-fit estimation for historical lineage dating, which could provide a very efficient and reliable way for genealogy and historical anthropology researches.

## Additional files

Additional file 1:
**Y chromosomal SNP haplogroups and STR data for pedigrees Shi and Cào.** The Y chromosomal SNP information and 17 Y-STRs (DYS19, DYS389I/II, DYS390,DYS391, DYS392, DYS393, DYS437, DYS438, DYS439, DYS448, DYS456, DYS458, DYS635, Y-GATA H4, and DYS385a/b) are provided. Additional file 2:
**The values of mutation rates applied in the calculations.** The evolutionary mutation rate (EMR), two observed genealogical mutation rates (OMRB and OMRS), and a genealogical mutation rate adjusted for population variation using logistic model (lmMR) are provided.

## Abbreviations

ASD: average squared distance
BATWING: Bayesian analysis of trees with internal node generation
EMR: evolutionary mutation rate
lmMR: genealogical mutation rate adjusted for population variation using logistic model
MCMC: Markov chain Monte Carlo
N: effective population size
OMR: observed genealogical mutation rate
SNP: single nucleotide polymorphism
STR: short tandem repeat
ya: year ago
